# A qualitative investigation of the factors influencing eating disorder symptomology during the postpartum period

**DOI:** 10.1186/s40337-025-01295-x

**Published:** 2025-07-06

**Authors:** Chantelle Ecob, Debbie M Smith, Zoe Tsivos, Sarah Peters

**Affiliations:** 1 Division of Psychology and Mental Health, School of Health Sciences, University of Manchester, Manchester, UK; 2 Greater Manchester Mental Health NHS Foundation Trust, Manchester, UK; 3 Manchester Academic Health Science Centre, Manchester, UK; 4 Division of Psychology and Mental Health, School of Health Sciences, Faculty of Biology, Medicine and Health, The University of Manchester, 2nd Floor Zochonis Building, Brunswick Street, Manchester, M13 9PL UK

**Keywords:** Postpartum, Eating disorders, Perinatal, Pregnancy, Maternal, Qualitative

## Abstract

**Background:**

The perinatal period can be a challenging time for women with current or historical eating disorder (ED) experience. Maternal EDs are associated with risks to both the mother and the child. During pregnancy, women are more likely to disengage with ED behaviours for the good of their growing baby. However, the postpartum period is a particularly risky period for the re-emergence or worsening of ED behaviours, irrespective of women’s pre-pregnancy ED status. Little is known about the factors which influence ED symptomology during the postpartum period. The aim of this study was to develop an understanding of the factors operating in the postpartum experience that influence ED symptomology.

**Methods:**

Semi-structured interviews were conducted with 12 women who had experience of an ED before becoming pregnant. Interview took place during or after the postpartum period, with women reflecting specifically on the postpartum period. Interviews were analysed using Reflexive Thematic Analysis.

**Results:**

Four themes were developed within the interview data; (1) *Embracing the self*, *(2) Motherhood: an ED enabler or protector?*,* (3) ED as a ‘plaster’ for emotional distress*,* and (4) The influential voices of others*. Theme 1 captures participants’ reports of the primary ED recovery facilitator, with a focus on self-awareness, self-understanding, self-compassion, and self-identity. Themes 2–4 describe a ‘triangle of powers’ which interacted with one another to influence participants’ ED symptomology both positively and negatively during the postpartum period.

**Conclusion:**

A new model is proposed which predicts that ED symptomology during the postpartum period is influenced by a complex interaction between various internal and external factors. Health care professionals encountering women with EDs during the perinatal period should be aware of these factors, to provide attuned and individualised care and improve outcomes for mothers and babies. Increased awareness how EDs may present during the postpartum period is needed.

**Supplementary Information:**

The online version contains supplementary material available at 10.1186/s40337-025-01295-x.

## Abstract (plain language summary)

The postpartum period (up to two years after giving birth) is a challenging time for women with experience of an eating disorder (ED). During pregnancy, a lot of women feel more able to reduce ED behaviours for the good of their growing baby. However, after they have given birth, many women with ED experience find that their ED symptoms reappear or become more frequent. We interviewed 12 women who had an ED before they became pregnant. We explored their experiences of the postpartum period, to understand the factors which made ED thoughts and behaviours more or less frequent with during this time. Despite an increased motivation to recover to feel like a better mother, women described various ‘powers’ which influenced their ED thoughts and behaviours, including loneliness and isolation, motherhood challenges and unhelpful messages from health care professionals, family, friends, and social media. In some cases, the ED was used as a coping mechanism. Self-awareness, self-understanding, and self-compassion supported women to move towards recovery and realign their priorities. The support systems around new mothers also play a vital role in improving ED outcomes during the postpartum period.

**Supplementary Information:**

The online version contains supplementary material available at 10.1186/s40337-025-01295-x.

## Background

Eating disorders (EDs) are mental health disorders characterised by severe and persistent disturbances in eating behaviour and related thoughts and emotions, that can significantly impair physical, psychological, and social functioning [1]. The Diagnostic and Statistical Manual of Mental Disorders [1] recognises eight different ED diagnoses, including Anorexia Nervosa (AN), Bulimia Nervosa (BN) and Binge Eating Disorder (BED). The estimated lifetime prevalence of EDs is 8.4% for women and 2.2% for men [2]. Eating disorders are accompanied by increased mortality rates [3] due to malnutrition, suicide, and physical issues, such as electrolyte imbalance [4]. Eating disorders are often chronic with low full recovery rates and high relapse rates [5].

Life cycle transitions (e.g., adolescence, motherhood and menopause), create shifts in individual’s sense of self, causing instability, confusion and anxiety [6] and are risks for the onset or exacerbation of ED symptoms [7, 8]. Though difficult to accurately assess due to lack of disclosure [9], research suggests that between 5 and 7.5% of women meet criteria for having an ED during pregnancy [10, 11]. Pregnancy, a time of increased contact with health care professionals (HCPs), provides multiple opportunities to support women’s mental health [12] and cross-system collaboration is recommended [13]. However, HCPs supporting women during the perinatal period report poor routine enquiry and knowledge about ED behaviours and symptoms [14, 15] and services have been described as fragmented, with limited opportunities for shared care [14]. Specialist ED services are also ill-equipped for perinatal support as no perinatal-adapted psychological treatments exist for EDs [13].

Compared with the general population, women with EDs are more likely to feel unhappy upon discovering their pregnancy [16]. Reasons include the prospect of weight gain, stress of eating for the baby and higher rates of unplanned pregnancies [17]. Patterns of ED symptomology vary during pregnancy. For some, their EDs remain stable throughout pregnancy [18] whilst for others symptoms worsen [19] or involve new ED symptomology, such as stopping laxative use but beginning to exercise excessively [20]. However, it is often the case that women are able to reduce or relinquish ED behaviours during pregnancy [21, 22].

Improvements in ED behaviours during pregnancy are often temporary with symptoms returning or worsening postpartum [22–25]. One study found that 12.8% of postpartum mothers were suffering with EDs, compared with 5.3% of prepartum mothers [26]. Makino, Yasushi & Tsutsui [27] found that 50% of women who were considered to be in ‘recovery’ from a previous ED prior to pregnancy, relapsed postnatally. Moreover, women in ‘recovery’ from an ED prior to pregnancy and those who continued to struggle with an ED during pregnancy, experience similar ED behaviours postnatally [24]. This suggests that the postpartum period can be triggering irrespective of how well women were coping with ED behaviours prior to becoming pregnant or during pregnancy.

Several factors that protect women from body dissatisfaction and ED-related thoughts during pregnancy, such as protecting the growing baby [21, 28], weight gain being directly linked to the developing foetus [29, 30], feeling the baby kick and increased view of body functionality [31], do not extend into the postpartum period. Postpartum women with EDs express feelings of loss of their pre-pregnancy bodies and a sense of urgency to ‘get it back’ [30, 32]. Women may feel they no longer have an ‘excuse’ for residual weight gain postpartum and the societal pressure to maintain the slim ideal returns [31].

The postpartum period is associated with increased depression and anxiety in women with any lifetime diagnosis of an ED, compared with women without a history of EDs [24]. Mothers with EDs often have greater concern for their child’s weight and eating habits [33]. They are more likely to report child temperament difficulties [34], eating problems [35, 36] and emotional disorders [37]. Mother-infant bonding may be impacted because they experience more communication and attachment difficulties [38]. Together this presents a need to understand the postpartum experience for women with ED, to ensure adequate community, medical and psychological support, and improve ED outcomes for the mothers, infants, and families.

There are gaps in the literature specifically pertaining to the postpartum period. Patel et al. [32] interviewed women without EDs, at risk for EDs and with current EDs, investigating their eating habits and attitudes postnatally. Women with EDs struggled to establish their new identity during the postpartum period due to loss of the pre-pregnancy self, life transitions, feeding relationship with their infant, new relationships with family members and new roles within wider society. However, data were collected using a formalised diagnostic interview [Eating Disorder Examination; 39], which provides limited information about the lived experience due to lack of interviewer flexibility and responsiveness to the participant [40].

Semi-structured interviews with women with EDs about their experiences of pregnancy and motherhood, reveal divided loyalties between motherhood and the ED, influenced by a fear of failure, transforming body, and eating behaviours, uncertainties about child’s shape and emotional regulation [23]. Women not meeting ED criteria were excluded from this study, meaning the voices of those able to maintain recovery postpartum were not explored. Researchers called for future investigation into factors predicting whether someone improves or relapses from an ED during the postpartum period. Thompson [41] began to address this gap by reviewing research into the trajectory of ED symptoms during the perinatal period. Possible postpartum-specific risk factors for ED symptoms were suggested, including socio-cultural pressure to ‘bounce back’ to pre-pregnancy body shape, a shift in women’s priorities towards body aesthetics over body functionality and increased body comparisons to their own pre-pregnancy body.

Given the risk of EDs worsening or re-emerging during the postpartum period, and the consequent risks to mother and baby, further qualitative research is required to explore the proposed risk and protective factors [23, 41, 42], including amongst women who were able to improve ED symptomology postpartum. The current study aimed to develop an understanding of the factors operating in the postpartum experience that influence ED symptomology.

## Methods

### Design

This qualitative study explored women’s experiences through in-depth semi-structured interviews, which were analysed using a critical realist approach to Reflexive Thematic Analysis [43].

Members of the public with lived experience of EDs and motherhood were invited to contribute to the study design via social media. Consequently, five individuals took part in a focus group, which was valuable in developing the language and terminology used within the research and the content of the interview schedule.

### Participant eligibility

Participants were eligible if they were UK residents aged 18 or over, had a historical or current ED (assessed through self-report), experienced at least one historical pregnancy resulting in a live birth, and provided contact details of a named clinician. Participants were excluded if they were currently pregnant or had experienced a stillbirth or neo-natal death.

### Recruitment

Participants were identified and recruited (March 2023 – March 2024) through NHS ED and therapy services in the Northwest of England. Clinicians circulated study information to clients who met inclusion criteria. Potential participants either provided verbal consent for the research to contact them or made contact themselves. Study information was also shared through social media, targeting relevant parenting and ED charities. To maximise diversity of ED diagnoses in the sample, the researcher also contacted support groups for specific ED diagnoses. Participants were sent a list of seven yes/no screening questions via email, and if eligibility criteria were met, they were emailed the Participant Information Sheet and Consent Form.

### Data collection

After electronically signing their Consent Form, participants completed a demographic questionnaire which recorded age, ethnicity, first part of postcode, year of giving birth, type of ED, whether this had been formally diagnosed, and contact details of a named clinician for risk management purposes. Participants were invited to take part in a semi-structured interview with CE, with the choice of face-to-face or via the online video platform Zoom.

Interviews were guided by an interview schedule developed collaboratively by the research team and PPI (Additional File 1), containing the following topics:

- Pre-pregnancy circumstances.
- Pregnancy and birth.
- Professional support.
- Social support.
- Socio-cultural factors.
- Relationship with baby.
- Physical health.
- Mental health.
- Critical periods.

Open questions and prompts were used to create conversations that invited narrative accounts to inform the research question [44]. The semi-structured nature of the interviews encouraged participants to describe their own personal experience, meaning the interview schedule was flexible, allowing exploration of relevant novel factors as they arose. Following the interview, a wellbeing check was completed and a £10 voucher was provided as a token of appreciation.

### Data analysis

Audio recordings were transcribed verbatim. Identifiable information was removed and pseudonymised transcripts were managed using NVivo 12. Data were analysed using Reflexive Thematic Analysis [43, 45], due to its open, exploratory, flexible, and iterative nature. Whilst the research objective was to explore women’s experiences of the postpartum period, the focus was on interpreting how these experiences interacted with their ED symptomology, which required a richer more nuanced understanding of the data [43]. Thematic analysis was employed as the research team aimed to capture a core, shared meaning across the dataset, beyond the obvious or surface-level content [40]. Braun and Clarke’s [46] adapted six stages were followed (Table 1). The process was collaborative, active and iterative throughout, with continual reference to the original data and the research question to validate and refine themes. Quotes that were illustrative of the themes and subthemes are presented in the results.

**Table 1:** Braun & Clarke’s (2022) adapted six stages of thematic analysis

Phase	Description of Process
Familiarising yourself with the data	This phase involves reading and re-reading the data, to become immersed and intimately familiar with its content, and making notes on your initial analytic observations and insights, both in relation to each individual data item (e.g. an interview transcript) and in relation to the entire dataset.
Coding	This phase involves generating succinct labels (codes) that capture and evoke important features of the data that might be relevant to addressing the research question. It involves coding the entire dataset, with two or more rounds of coding, and after that, collating all the codes and all relevant data extracts, together for later stages of analysis.
Generating initial themes	This phase involves examining the codes and collated data to begin to develop significant broader patterns of meaning (potential themes). It then involves collating data relevant to each candidate theme, so that you can work with the data and review the viability of each candidate theme.
Developing and reviewing themes	This phase involves checking the candidate themes against the coded data and the entire dataset, to determine that they tell a convincing story of the data, and one that addresses the research question. In this phase, themes are further developed, which sometimes involves them being split, combined, or discarded. In our TA approach, themes are defined as pattern of shared meaning underpinned by a central concept or idea.
Refining, defining and naming themes	This phase involves developing a detailed analysis of each theme, working out the scope and focus of each theme, determining the ‘story’ of each. It also involves deciding on an informative name for each theme.
Writing up	This final phase involves weaving together the analytic narrative and data extracts, and contextualising the analysis in relation to existing literature.

Note. Adapted from https://www.thematicanalysis.net/doing-reflexive-ta/

### Rigour

To ensure that themes were ‘trustworthy’ and grounded in the data, the approach to analysis was iterative and reflexive [47]. The first two authors independently coded the first two interviews, to ensure their interpretations of participants narratives were similar and consistent with the data. All four authors read each transcript and the first author took a lead role in coding the subsequent transcripts. Researcher triangulation [48] was practiced by the research team. This process supported a shift from themes as summaries of topics, towards interpretative stories built around uniting meaning [46].

### Reflexivity

As qualitative research is influenced by researcher experiences, preconceptions, and knowledge [49], it was crucial to consider the subjective positioning of the research team. The research team comprised a Trainee Clinical Psychologist (CE), two Health Psychologists, both with expertise in qualitative and pregnancy research (DS & SP), and one Clinical Psychologist working within an NHS ED Service with a special interest in the perinatal period (ZT). A reflective log was held by the lead researcher (CE) and ongoing discussions within the research team supported the reflexive process [50].

Two members of the research team were parents (DS & SP), and two were non-parents (CE & ZT). This supported the team to adopt an ‘in-between’ positionality located somewhere on the ‘insider-outsider’ continua [51]. As no members of the research team had personal experience of having an ED, the public involvement in study design was crucial.

## Results

### Sample characteristics

There was an initial influx of interest, which gradually slowed and recruitment became increasingly difficult. In total, twenty-one women expressed an interest in participating, of which four did not return their consent form, three did not meet eligibility criteria (one was currently pregnant and two developed their ED after the birth) and two were not interviewed for ethical reasons (due to pre-existing personal relationships). Interviews averaged 63 min (45–75 min) and were conducted on Zoom (*n =* 11) or face-to-face in a private room at a community centre (*n =* 1). Recruitment concluded after 12 interviews due to a collaborative decision that data with sufficient information power [52] relevant to the research question had been collected [43].

Participants were recruited to the research from various streams; social media (*n* = 5), mental health services (*n* = 4), a specialist support group (*n* = 2) and an ED charity (*n* = 1).

**Fig. 1:**
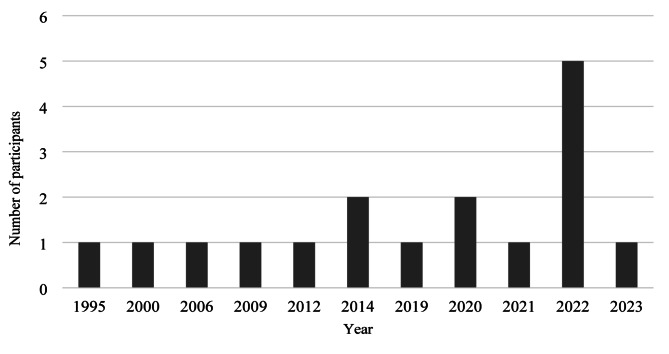
Years participants gave birth

**Fig. 2:**
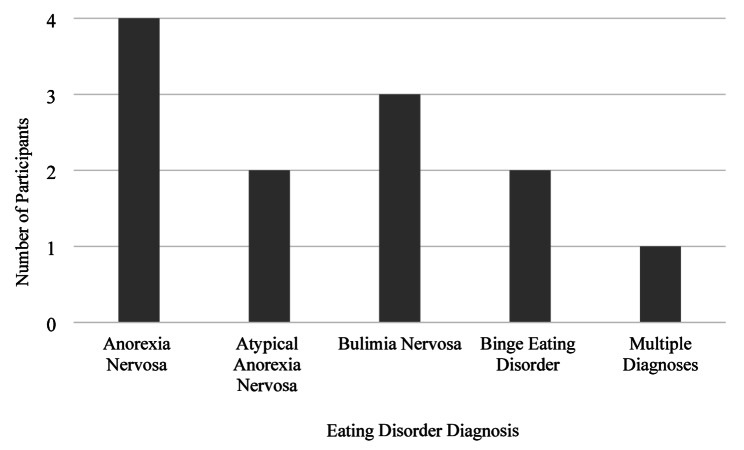
Formally or self-reported ED diagnoses of the 12 participants Note: The participant under the Multiple Diagnoses category reported diagnoses of AN and ARFID

Participants were aged between 25 and 62 (*M = 39 years*,* SD* = 10.8) and were White British (*n* = 11) or White Other (*n* = 1). Six participants resided in the Northwest of England, two in the East Midlands, two in the Southeast of England, one in the East of England, and one in the Lothian region of Scotland. Seven women had given birth once, and five women had given birth twice. In the latter case, women shared their experience of both postpartum occasions. Time since giving birth ranged from one–28 years (*Mode* = two years). See Fig. 1 illustrating the years participants gave birth. Ten participants reported that they were formally diagnosed with an ED by a HCP, and two women described themselves as self-diagnosed (See Fig. 2 for ED diagnoses).

### Findings

The findings are presented as a thematic structure comprising four themes. The first theme, *(1) Embracing the self*, encapsulates participants’ reports of the primary ED recovery facilitator, where they were able to focus on understanding the ED, expanding their sense of identity, and developing self-compassion, in some cases in the context of psychological therapy. The other three themes, *(2) Motherhood: an ED enabler or protector?*,* (3) ED as a ‘plaster’ for emotional distress*,* and (4) The influential voices of others*, describe a triangle of interacting powers that participants described experiencing during the postpartum period. These three powers were described by participants as both barriers and facilitators to recovery, dependent on how they were engaged with.

**Fig. 3:**
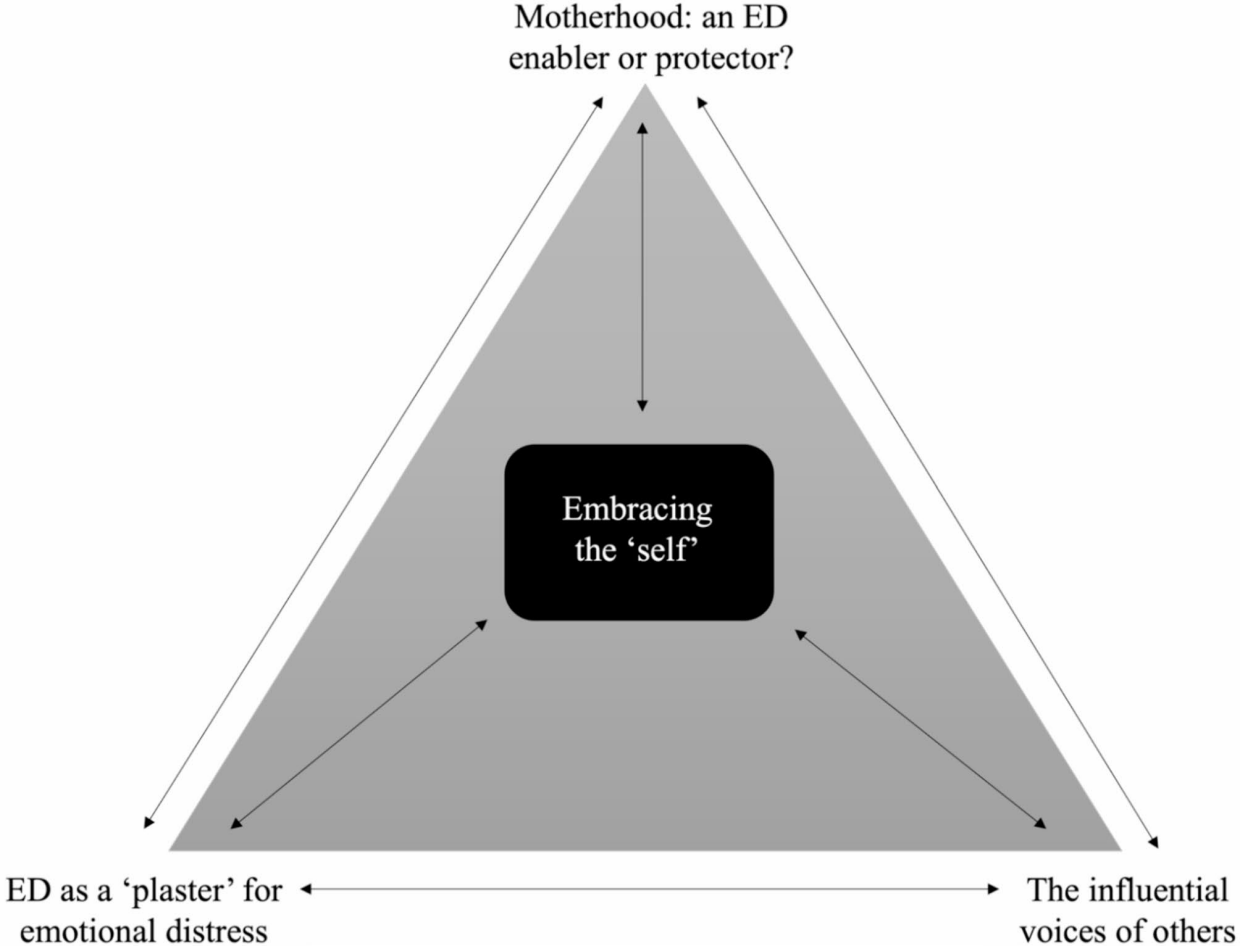
Thematic diagram summarising the four themes

The themes are described below with quotes from interviews and corresponding ID numbers. Figure 3 presents a thematic diagram demonstrating how the four themes interact with one another to influence participants’ ED symptomology.

### Theme 1: embracing the ‘self’

Participant’s narratives revealed the importance of connecting with and valuing themselves as an individual with their own identity, which was described as promoting ED recovery postpartum. This theme sits in between the triangle of powers, as it was described to be heavily influenced by the other three themes in both positive and negative ways. Participants who felt more able to recover from their ED during the postpartum period all discussed the importance of pre-pregnancy self-awareness and coping skills.

*I think because I*,* I knew that it could be a trigger*,* I was like alright*,* if that makes sense? Like I knew*,* okay*,* your body is going to change. You’ll have to accept that (Participant 7)*.

Participants who sought ED-focussed treatment valued learning to recognise their ED thoughts, thinking patterns and maintenance factors, learning to reframe these thoughts and hold alternative perspectives. Participants reported that when they were preoccupied with ED thoughts, this indicated that they might be worrying about something else. Understanding the ED and learning to recognise this pattern allowed participants to ‘detach’ from their ED and diffuse the associated thoughts and behaviours. Participants found it helpful to learn how their ED impacted upon their body and to receive compassionate nutritional education alongside *“unconditional permission to eat” (Participant 12).* Participants who described having increased self-awareness and understanding of their ED felt more prepared for and better able to cope with the potential postpartum triggers, including being able to ignore unhelpful and potentially triggering advice.

*It’s made me understand the eating disorder…About the effects it can have on my body*,* and why I’m feeling fat rather than bloated. And yeah*,* helped me understand some of the complications*,* some of the viewing styles of eating disorders. In terms of like black and white thinking. (Participant 11)*

*So when I was overeating but equally when I was undereating*,* it was those anorexic thoughts of I don’t deserve to eat. And I can still have those thoughts*,* but I recognise them now. I have to take myself out and stop. (Participant 9)*

Other participants spoke about engaging in more general psychological therapy, not specific to their ED, which increased their understanding of why the ED initially developed as a product of adverse childhood experiences, allowing increased self-compassion, and feeling exculpated. This reduced feelings of guilt and shame, and negated the need to use the ED as a coping mechanism for overwhelming distress. Two participants described the importance of compassionate self-talk, and the value of learning that eating in a loving way indicated emotional stability. Learning to be a *“good enough” (Participant 11)* mother was described as reducing the ED perfectionism voice.

*Just learning*,* a lot of the therapy was kind of like inner child work*,* and learning to just show compassion to that little girl…So I think a lot of it was just about more compassion for myself. More understanding. Yeah*,* it made a big difference. (Participant 4)*

Alongside developing self-awareness and self-compassion, participants valued widening their sense of identity beyond the scope of ‘mother’ and ‘ED’. It helped to see the *“eating disorder brain as a separate thing” (Participant 12)*. This required an ability to distinguish ED thoughts and identify them before acting on them. Returning to work and socialising also helped re-develop self-identity and gain a sense of achievement beyond the ED.

*I do have different things that I feel the sense of achievement for. Now I suppose one of them is my business working with people. So you know*,* that’s motivational. (Participant 7)*

### Theme 2 – Motherhood: an ED enabler or protector?

One factor within the overall triangle of powers was participants’ new context of motherhood. Motherhood presented opportunities for participants to recover from the ED, as well as aspects which enabled, maintained or worsened the ED. This theme is organised into two subthemes.

#### Subtheme 2.1: motherhood making ED easier to hide and thrive

Despite having more contact with HCPs, family and friends, participants found that the focus of this support shifted from them towards the baby, leaving them feeling isolated. Postpartum isolation provided an opportunity for the ED to hide and thrive. For example, mothers with AN described the busyness of motherhood provided the perfect ‘excuse’ for skipping meals, and mothers with BED found hiding their binges easy due to maternity leave and social isolation.

*I didn’t have support*,* and I didn’t feel able to talk to my friends about it*,* because*,* well people stop asking. They’re just asking how you are as a mum. How’s the baby? No one’s asking you*,* how is your eating disorder anymore. Because that’s fixed because you had a baby. (Participant 2)*

*I would seek out being alone. I mean*,* alone in the car*,* or*,* you know*,* sort of alone at home*,* or something like that. And I would eat and eat and eat and eat and eat. (Participant 10)*

Participants reported an increased desire to breastfeed to burn calories and lose weight, which consequently gave power to their ED voice. Some felt pressured by HCPs, family members, online messages and other mothers to breastfeed. Breastfeeding difficulties led to a double sense of guilt due to not burning the calories, and not feeding their baby ‘correctly’.

*I think there’s a lot of societal pressure to breastfeed. And for me I just felt like there was an added guilt of not burning those extra calories that I found hard as well. (Participant 5)*

Participants found their postpartum body become a target of unhelpful attention and *“public property” (Participant 2)*. They felt mixed emotions when praised for postpartum weight loss, by HCPs, friends and complete strangers. Some reported a sense of achievement because others were *“complimenting the eating disorder” (Participant 12);* whereas some felt a sense of invalidation as weight loss was caused by ED behaviours. Importantly, irrespective of the feelings this ignited in mothers, they often served to maintain ED behaviours.

#### Subtheme 2.2: managing an internal fight: baby vs. ED

Motherhood caused a shift from a dyadic relationship between the woman and the ED, to a new relationship incorporating the baby. This created a conflict between the demands of the baby and the demands of the ED, which impacted the course of their ED in various ways.

Participants reported increased self-consciousness and hypervigilance to their postpartum bodies, wherein their appraisal of changes, such as increased weight and loose skin around the stomach, influenced ED symptomology. Participants described feeling like their postpartum body changes were *“unjustified” (Participant 2)* as the baby was no longer in there. These feelings increased ED behaviours, negatively impacted on intimate relationships, and discouraged social interaction. However, some participants could shift their priority from the ED to the baby and described feeling more forgiving of postpartum body changes because without them, the baby would not exist.

*The hardest thing for me was my body*,* and accepting that my body had changed. But so had my life…And without it*,* I wouldn’t have had this little beautiful human. (Participant 3)*

For some mothers, the addition of the baby provided a new perspective on what was important to them, and encouragement to ‘do better’ for the good of their baby. For others, the shift from ED to baby was more about being too busy to maintain the ED, or fear that something bad would happen to their baby if they engaged with the ED. This reprioritising supported many to recover.

*I think it is I just can’t make working out the priority*,* because then I would have to be ignoring her…I haven’t weighed myself after my daughter*,* because*,* like it just doesn’t matter anymore. (Participant 7)*

Being a role model for the baby was described by several participants, who were motivated towards recovery by a fear of *“passing on” (Participant 4)* the ED by observing and imitating their behaviour. These fears were particularly focused on female rather than male infants.

Participants reported feeling like a failure of a mother due to their ED. Feelings of guilt, shame and worry were common. The feeling of shame caused by ED struggles postpartum was described by some mothers as a motivator to recover, whilst some found that the shame contributed to a vicious cycle of emotional distress and increased ED behaviour as a coping mechanism. Participants described a difficult fight for control between the ED and motherhood. The ED was described as a constant *“white noise” (Participant 9)* which was battling with maternal thoughts of protecting the baby. Some participants described the ED as being completely in control, despite a strong desire to recover, which kept them stuck in the vicious cycle.

*There was like a question of*,* well*,* yes you’re producing milk but are there the right nutrients in it? Like*,* is he getting his needs met? And so I*,* I just felt like sick with shame about the whole thing. I was like*,* I starved him in the womb and I’m starving him now. (Participant 6)*

Where the ED was more powerful than the maternal urge to recover for the baby, participants described using the baby’s good health as a justification to continue their ED behaviours. For example, if participants were not experiencing breastfeeding difficulties and their babies were growing as expected, this removed the justification for food and the ED retained control.

### Theme 3 – ED as a ‘plaster’ for emotional distress

The ED was described as a *“plaster” (Participant 1)* for emotional distress. In this respect, the word ‘plaster’ (also known as a band-aid) was used as a metaphor to represent a temporary coping mechanism. Participants described feeling comforted or distracted by their ED, negating a need to process and address difficult emotions. The need for this ‘plaster’ was particularly important where participants felt stuck in the cycle of shame and emotional distress described above, which maintained ED symptomology.

*You know it was*,* it was reaching for my drug of choice*,* because I was desperately avoiding feeling the emotions*,* and I was desperately avoiding dealing with any of the big issues. (Participant 10)*

Two subthemes were developed within this theme.

#### Subtheme 3.1: ED providing structure and control

The ED provided some participants with a sense of structure and control, where the new demands and uncertainty of motherhood stripped them of this. These participants reported that their ED provided them with control over their food and weight, and gave them something else to focus on, where so much felt out of their control. For some, they feared the postpartum period presented a *“slippery slope of falling back into bad habits” (Participant 8)*, such as overeating and not exercising. The ED provided a set of rules to cope, and the ease of returning to their pre-pregnancy eating structure was described.

*I remember weighing myself*,* I think he was 2 or 3 weeks old*,* and I weighed myself*,* and I just saw the number*,* and I was like*,* no*,* this can’t be happening. I can’t be going through all this*,* and that’s my weight. I need the weight to come down. I can control that. And I think that’s sort of what really tipped me into using restriction again. (Participant 5)*

#### Subtheme 3.2: the loading of adversity

Participants shared many experiences of trauma or adversity. Childhood traumas were recognised as important in shaping the conditions for developing an ED. Self-reported traumatic birthing experiences were described by four women, who felt that this ‘loading’ of adversity directly worsened their ED. Increased adversity led to an increased need to utilise the ED as a ‘plaster’.

*It was a trauma to be honest*,* and it was a trauma originally that kind of triggered my eating disorder I guess. So that*,* it was like a new trauma and I didn’t know how to*,* how to deal with it. How to cope. (Participant 3)*

Experiences of post-natal depression were also prevalent, which caused feelings of guilt and increased the need to utilise the ED as a ‘plaster’. Post-natal depression was described to cause a mental *“shutdown” (Participant 4)* and self-neglect through starvation.

### Theme 4 – The influential voices of others

Participants described the positive and negative impacts of outsider voices on their postpartum experiences. Within this theme, the ‘other’ refers to anyone outside of the mother-baby relationship, including HCPs, health services, friends, family, and social media. These voices were described as powerful and highlighted the vulnerability and susceptibility of the ED during the postpartum period. Three subthemes were developed within this theme.

#### Subtheme 4.1: misattuned professional support – a gap in the system?

All participants spoke about their experience of support from HCPs postpartum. Participants described being passed between ED services and perinatal mental health services, with each stating that they were not equipped with the knowledge to support this overlap. Perinatal mental health services were described as focusing on general mental health and risk, rather than EDs. Participants encountered a lack of both specialist perinatal knowledge within ED services, and a lack of specialist ED knowledge in perinatal services. As a result of this gap in the system, advice could unintentionally trigger, enable, or maintain ED behaviour.

*I was admitted that day to the MBU. And it was awful because no one there knows anything about eating disorders. I remember on the first day I was there*,* one of the nurses said “oh you’re lucky*,* we don’t normally let people with eating disorders in cause it’s not really a serious enough illness.” (Participant 6)*.

Participants described misattuned support from HCPs, where they felt their ED was missed, dismissed, or minimised. Participants reported feeling like their ED signs and symptoms, such as weight loss, weight gain or general exhaustion, were treated like the *“elephant in the room” (Participant 9)* or *“brushed under the carpet” (Participant 3).* Participants felt like the signs were *“obvious” (Participant 10)* and hypothesised that HCPs are *“too scared” (Participant 1)* to acknowledge them. Health care professionals’ investigations into participants’ mental health were viewed as a *“box ticking” (Participants 2*,* 8*,* 12)* exercise translating into little support. Some participants who did chose to disclose their ED to a HCP reported feeling invalidated or ridiculed, which served as an enabler for their ED to continue into the postpartum period as the seriousness of the situation was minimised.

*The hospital where I was delivering tried to refer me to a Dietitian*,* and she was like “don’t worry*,* when you have the baby you’ll be too busy*,* and all these selfish thoughts won’t be there anymore”. (Participant 2)*

#### Subtheme 4.2: the dilemma of sharing the distress

Participants found it difficult to disclose their ED to others during the postpartum period due to feeling ashamed, fearful of disappointing others, undeserving of support, minimisation, and avoidance of the ED, feeling vulnerable and finding it difficult to relinquish control. Participants were only able to access support when they had acknowledged their ED.

The ED was described as a way of communicating distress. Partly this was feeling unable to permit themselves to recover, instead seeking this justification from others. Participants were relieved when HCPs recognised their ED, and allowed them to follow recovery-based advice, which they felt unable to take from themselves.

*I think part of my eating disorder has always been that I find it really hard to ask people for help*,* or to say that I’m struggling. And so to see that I’m visibly kind of fading away. It’s a way of like showing people that I’m struggling when I can’t find the words to do it. (Participant 1)*

*And she kept giving me permission to eat. She kept saying*,* it’s okay to eat*,* because I think she picked up early on that I really struggled with giving myself permission to eat*,* more than anything. And I think that was a huge thing. (Participant 12)*

Participants described the helpfulness of receiving accepting and non-judgemental responses from others. Having their ED *“out in the daylight” (Participant 4)* reduced secrecy and shame around their behaviours. With less shame and isolation, participants felt more able to work towards recovery. Some also reported increased accountability after disclosing their ED, which increased motivation to recover.

*So much of it is all about secrecy and lying to people. So I would force myself. I went through a period of time where I*,* when I was making myself sick*,* where I would tell him [partner] after I’d done it. Just to kind of force it out in the open. Erm*,* partly to remove shame*,* but also*,* you know*,* just to make it more open so I wasn’t hiding away. (Participant 4)*

Participants described feeling valued when HCPs took time to understand their individual ED experience and demonstrate a shared motivation towards recovery, which allowed them to accept alternative perspectives and *“reality checks” (Participant 11)*, as well as encouraging them to *“commit to trying harder” (Participant 1).*

*Definitely my midwife was like instrumental*,* because she just said to me when I told her I had anorexia she went*,* I’ve never had someone with anorexia before. She was like*,* I’m going to go and spend my evening reading about it. And like*,* you know*,* that she cared that much was really nice. (Participant 1)*

Sharing difficulties with other women with EDs helped women feel understood, less alone, and hopeful about recovery.

*It’s that feeling that people understand you. Hook line and sinker*,* because they have done what you have. You hear people’s stories and they are no longer doing what you’re doing*,* and you really think*,* well if they can do it*,* I can do it. (Participant 9)*

Another benefit of disclosing the ED was accessing practical support, which helped support ED recovery. For example, participants valued having food prepared for them, or being prescribed a meal plan by their dietician, which promoted recovery-focussed behaviours. Help from partners and family members with household tasks and childcare reduced exhaustion and emotional distress, negating the need to utilise the ED ‘plaster’. This required participants to relinquish some control, which was difficult as participants reported increased need for control postnatally.

#### Subtheme 4.3: social pressure & comparison with others

Participants felt increased pressure from social media advertisements and interactions with other new mothers to breastfeed, follow postpartum diet plans and return quickly to a pre-pregnancy body shape.

*So there was this online mum forums. And everyone was posting stuff like*,* oh yeah I’m going to join slimming world*,* I’m going to shift those pounds that I’ve gained. Which for me was like*,* oh well*,* this is what I need to do. The pressure from society to bounce back is extraordinary. (Participant 3)*

Some sought nutritional advice on the internet, which focussed on weight loss and presented mixed messages about what they should and should not be eating. Participants described unrealistic portrayals of women and motherhood which set unrealistic standards that participants felt obligated to meet, due to their perfectionistic nature.

*I would say if you look on Instagram*,* and there’s someone like*,* oh I’ve got my pre-pregnancy body back in a week*,* and I eat these things. And I look and think I should do that. (Participant 7)*

Participants worried their ED impacted how they were viewed as a mother, with the ultimate fear that their baby would be removed. For some, this fear prompted them to recover from their ED. However, for others, this fear stopped them from sharing their ED difficulties, which created further shame and secrecy and maintained ED behaviour as a coping mechanism.

*It was like a week after that*,* my care co-ordinator came to see me*,* and she said that they were very close to referring me to social services because they were worried that I wasn’t fit to take care of [baby’s name]. Erm*,* which then*,* that prompted me to end the relapse because that destroyed me. (Participant 5)*

## Discussion

This is the first study to qualitatively investigate women’s experience of the postpartum period, where they had an ED prior to pregnancy. Women’s accounts reinforced that the postpartum period is a challenging and sensitive window, with variability in ED symptomology. The current study proposes a new model, which summarises a triangle of powers acting on women during the postpartum period, which were described to influence ED symptomology variably. Central to the proposed model is women’s ability to embrace the ‘self’, and exercise self-awareness, self-understanding, and self-compassion amid the interplay between the various powers present during the postpartum period.

The triangle of powers expands the biopsychosocial model [53] of EDs, which suggests that key factors in the development of EDs include sociocultural pressures to be thin, body dissatisfaction, low self-esteem, environmental stressors, cognitive distortions, and inadequate identity formation [54]. These factors are heightened during the postpartum period, where new mothers experience an increased societal pressure to ‘bounce back’ to pre-pregnancy body shape (subtheme 4.3), experience new stressors associated with motherhood (theme 2) and are highly vigilant to perceived judgement from others (theme 4). Additionally, women described a battle with their ED voice during the postpartum period (subtheme 2.2). Perceived benefits included its efficacy as an emotional ‘plaster’ to protect against emotional distress, and perceived costs included increased feelings of guilt and shame. This created a vicious cycle where the perceived costs can be managed by the perceived benefits. This highlights the importance of the third power, the influential voices of others (theme 4). The data revealed the potentially precarious nature of participants reaching out and accepting support from others (subtheme 4.2), where in some instances it can facilitate ED recovery, but in other circumstances it can enable or worsen ED symptomology. This presents a difficult ‘tightrope’ for others to walk and highlights the importance of trusting relationships, attunement, and individualised support.

Participants’ experiences of being pulled into the triangle of powers during the postpartum period could be explained by Attachment Theory [55]. Individuals with EDs are more likely to have an insecure attachment pattern [56, 57], characterised by a dilemma between compulsive care-seeking (anxious) and compulsive self-reliance (avoidance) [58]. Women’s narratives in the current study demonstrated the dilemma of sharing the distress (subtheme 4.2), where reaching out and accepting support was described as both challenging and vulnerable due to the ED voice, as well as a strong ED recovery facilitator. Forsén Mantilla, Clinton & Birgegård [59] suggest that individuals with EDs may experience their ED as an attachment relationship, within which it provides an ‘unsafe haven’ which they may seek proximity to during times of heightened distress and disarray. The current findings suggest that this may be due to the ED providing structure and control (subtheme 3.1). Times of heightened vulnerability and need, such as the birth of a child, activate the attachment system [55], suggesting that the transition to motherhood and the new mother-baby relationship may place increased threat on the woman-ED relationship. Subtheme 2.2 suggests that new mothers with previous or current EDs experience an internal fight between returning to the security of their ED relationship and investing in a new attachment relationship with their baby. Women’s narratives revealed an important protective ability to develop self-identity and view the ED as a separate entity (theme 1). Narrative therapy [60] uses techniques centred on externalising the ED and understanding the nature of the relationship between person and disorder, which may be particularly helpful during the postpartum period with the opportunity for a new attachment relationship for women to invest in instead.

### Clinical implications

Patel et al. [32] found that women with EDs were more likely to perceive the external world as more negative and critical in the postpartum period, than women without EDs. The current study found that women with ED experience often perceived professional support as misattuned (subtheme 4.1) and experienced HCPs as purposefully dismissing and invalidating, which may be compounded by repetitive negative thinking patterns often experienced by individuals with EDs [61]. Additionally, HCPs working with individuals with EDs often experience frustration, helplessness, and distress due to support being unwanted or insufficient, which can lead them to ‘block it out’ or blame service-users for their difficulties [62]. Health care professionals working with women with ED experience during pregnancy and postpartum (including GPs, Midwives, Health Visitors, Obstetricians, Dieticians, Psychiatrists and Psychologists) should be aware of their own emotional experiences, to monitor transference and countertransference processes which may be harmful if not managed appropriately [63], particularly during the postpartum period where their voices are highly influential (theme 4) and more sensitive to perceived criticism. This may be supported by clinical supervision, which is recommended for HCPs working with EDs and perinatal frontline staff [64, 65].

Contrary to previous literature suggesting that once women with EDs are no longer concerned about their health “for the baby”, they tend to lose their drive to stay healthy for themselves [31], theme 2 demonstrates that motherhood acted as both an ED enabler and protector, where some women were able to maintain the motivation to take care of their bodies for the baby after birth, partly by acknowledging that their health still had a direct impact on the baby via breastfeeding, parenting ability and role modelling. This difference may be mediated by self-objectification [66], where there are overvalued ideas around weight, shape and appearance over body function, which is linked with increased disordered eating during the postpartum period [67]. It may be helpful for HCPs to advise women about their body’s ongoing functionality during the postpartum period, and normalise and validate postpartum body-aesthetic changes, to maintain the protective factor of body-functionality post-pregnancy and enhance the protective mechanisms of motherhood (theme 2). Whilst positive body image has been identified as an ED protective factor [68], researchers have questioned whether any emphasis on body-aesthetics may continue to reinforce a preoccupation with appearance [69]. As women with ED experience describe increased social pressure and comparison with others during the postpartum period (subtheme 4.3), the novel concept of ‘body neutrality’, which promotes neither positivity nor negativity towards the body, but rather acceptance of and respect towards it [70], may be a more helpful and realistic goal.

Participants’ narratives highlighted the ease of returning to pre-pregnancy eating behaviours and structure after giving birth. This supports the notion of pregnancy being a window of opportunity for psychological intervention, where motivation is increased and there is a temporary change in behaviour [71]. Sharing the distress (subtheme 4.2) and developing a more accepting, understanding, and compassionate sense of self (theme 1) were described as promoting ED recovery postpartum, suggesting that women with ED experience would benefit from psychological therapy during the perinatal period to create shifts from initial guardedness to openness, self-blame to self-compassion, dismissiveness to self-validation, and from the perfect to good enough mother [72]. There are currently no psychological treatments for EDs which are adapted for the postpartum period [13] and current treatments may lack the depth and attention to the triangle of powers at play during the postpartum period. Psychological treatments for postpartum EDs should be tailored to the additional needs of women during the postpartum period.

The findings of the current study identified increased social comparison during the postpartum period, which often maintained ED symptomology. Social comparison is associated with various negative maternal outcomes, including parenting, relationship, and mental health difficulties [73]. Self-compassion appears to buffer the association between self-comparison and ED symptomology among postpartum women [74] and was described by the current sample as supporting them to successfully shift their priority from the ED towards motherhood (theme 1). Women seeking ED treatment postpartum may benefit from a treatment which enhances self-compassion, such as compassion-focussed therapy for EDs (CFT-E) [75] which aims to address shame and self-criticism and to assist patients in developing greater self-compassion. Early research demonstrates promising outcomes [76, 77], including for those with a history of trauma [78] which was described by the current sample as another compounding factor in their postpartum experiences (subtheme 3.2). However, no studies to date have evaluated the use of CFT-E during the perinatal period.

### Strengths and limitations

As demonstrated within the findings, perinatal EDs are associated with increased levels of shame and fear of negative judgement from others, which may have contributed to recruitment difficulties in the current study. Despite a relatively small sample size, the interviews were in-depth and demonstrated high information power [52], supported by a sample with variation in age and ED diagnosis. Much of the current literature on perinatal EDs focuses on restrictive EDs, such as AN and BN, with a lack of research into BED [29]. Importantly, the findings indicated that the despite the symptomology of BED, AN and BN differing, the postpartum experiences were similarly influenced by the themes described. Although participants described variation in ED symptomology prior to pregnancy, their pre-pregnancy ED ‘status’ was not quantitatively measured. This might have been a useful tool in identifying patterns in the data. However, research suggests that women in ‘recovery’ from an ED prior to pregnancy and those who continued to struggle with an ED during pregnancy, experience similar ED behaviours postnatally [24]. The current findings support this as women’s pre-pregnancy ED symptomology was not described as a key factor in determining postpartum ED symptomology.

The sample lacked ethnic diversity because all participants in the current sample identified as White British or White Other. Ethnic minority women have lower access to community mental health services during the perinatal period [79] and report poorer experiences of perinatal mental health care due to stigma, language barriers and culturally insensitive services [80]. This suggests that women from ethnic minorities with EDs may experiences alternative or additional powers impacting ED symptomology during the postpartum period. Future research should focus on the role of ethnic diversity in ED symptomology during the postpartum period. Another potential limitation of the study is that the eligibility criteria did not limit the length of time between birth and interview date. As demonstrated in Fig. 1, the length of time between birth and interview date was variable, and some participants discussed experiences from up to 28 years ago. This increases the risk of recall bias within interviews and may question the validity of the findings. Recall during interviews was not recognised as a problem, perhaps because memories containing intense emotions are more vividly remembered [81]. The length of interviews, recollection of details and richness of the current data demonstrates the pertinence of the postpartum period for participants.

### Future research recommendations

Women’s narratives emphasised the important roles of HCPs within their postpartum journey, with a common subtheme of misattuned support enabling or maintaining ED symptomology. It is noted that the voices of HCPs were not explored within the current study. 85% of health visitors report staff shortages, and 86% report that there is not enough capacity in other services to pick up onward referrals [82]. Additionally, both midwives and health visitors have described a lack of opportunity and time to enquire about EDs within routine antenatal appointments [14]. Health care professionals working with patients with EDs commonly report limited knowledge and training and increased feelings of frustration, boredom, helplessness and emotional exhaustion [14, 83] and this could be amplified by the lack of contact that perinatal HCPs (e.g., midwives, health visitors, obstetricians) have with women with EDs within their overall caseload. It may be derived that HCPs feel unable to provide adequate support for women struggling with EDs postpartum, due to workforce shortages, lack of training and emotional burnout. It is recommended that future research explores the experiences of HCPs supporting women with current or previous EDs during the postpartum period, to identify potential barriers to support.

To make the findings of the current study more transferable to practice, future directions for research include further examination into the protective factors proposed. Since the voices of others have been found to be influential, it would be helpful to develop an understanding of how non-professional support systems around new mothers, such as family and friends, might support ED recovery postpartum. Further exploration of the protective role of ‘embracing the self’ will be important, including the factors which might make women more or less able to exercise self-awareness, self-understanding and self-compassion during the postpartum period.

## Conclusions

The current study offers a novel insight into the complex interplay between various factors influencing ED symptomology during the postpartum period, where women had previously suffered with an ED. The postpartum period is a risky and vulnerable period for ongoing or re-emerging ED symptomology. The transition to motherhood can enhance women’s motivation to recover from their ED, whilst also providing circumstances enabling ongoing ED symptomology. The ED may be used as a ‘plaster’ to protect against increased emotional distress postpartum, where there is an increased focus on social comparison and the voices of others become more influential. Embracing the ‘self’ and building self-awareness, self-understanding and self-compassion are protective mechanisms amongst this triangle of powers which cause a push-pull dynamic between new mothers and EDs. The support systems around new mothers play a vital role in improving ED-related outcomes during the postpartum period.

## Electronic supplementary material

Below is the link to the electronic supplementary material.

Supplementary Material 1

## Data Availability

No datasets were generated or analysed during the current study.

## Abbreviations

ED: Eating disorder
AN: Anorexia nervosa
BN: Bulimia nervosa
BED: Binge eating disorder
PPI: Patient and public involvement
HCP: Health care professional
