# Supplementary material for: A qualitative investigation of the factors influencing eating disorder symptomology during the postpartum period
Source: J Eat Disord. 2025 Jul 6;13:127. doi: 10.1186/s40337-025-01295-x (PMC12232848; doi:10.1186/s40337-025-01295-x)
Supplement: Supplementary file 1 — Supplementary Material 1 [file 40337_2025_1295_MOESM1_ESM.docx]

**Additional File 1**

*Interview Schedule*

| **Broad topic** | **Open questions** | **Prompts** |
| --- | --- | --- |
| **Pre-pregnancy circumstances**  *(Eating disorder status, feelings about pregnancy, preparedness)* | - How would you describe your pre-pregnancy circumstances? - How would you say you were coping with your eating disorder prior to becoming pregnant? - What were your initial thoughts when you found out you were pregnant? - How would you describe your life circumstances at this time? | - How did you feel about that? - How did you think it was going to go? - What were your worries/concerns? - What about social support? |
| **The pregnancy and birth**  *(Eating disorder symptoms, level of support, delivery of baby)* | - How would you describe your pregnancy? - How would you say you coped throughout your pregnancy? - How supported did you feel throughout your pregnancy? - How was your experience of the birth? - Would you say your pregnancy and/or birth had any impact on your experience of the postpartum period? | - Could you tell me a bit more about this? - How do you feel this affected you during the postpartum period? |
| **Professional support**  *(Interactions with health care professionals, access to support, quality of support, language used by HCPs)* | - How would you describe the support you received from professionals? - How well you would say you were supported by health care professionals in the postpartum period? - How would you describe your relationship with your midwife/health visitor? - If you could change the support you were given, how would do this? - How, if at all, do you think this impacted on your eating disorder? | - Was there anything particularly triggering about this? - Was there anything particularly helpful about this? - Is there anything you know now that you wish you knew then? - Is there any further support you wish you had access to? - How do you feel this affected your thoughts, feelings and behaviour? - Can you think of any specific examples of interactions which felt influential? |
| **Social support**  *(Family, partner, friends, isolation)* | - How would you describe your social support during the postpartum period? - How , if at all, do you think this impacted on your eating disorder? | - How did this feel? - How do you feel this affected your thoughts, feelings and behaviour? - Can you think of any specific situations or examples? |
| **Socio-cultural factors**  *(Cultural or religious influences, social media, pressure to ‘bounce back’)* | - Do you feel your cultural or religious views impacted on your experience of the postpartum period? - Do you feel your cultural or religious views impacted on your eating disorder during this time? - Did you feel any societal pressures during this period? | - How do you feel this affected your thoughts, feelings and behaviour? - What specific views/traditions/messages felt influential during this time? |
| **Relationship with baby**  *(Early attachment/bonding, feeding)* | - How would you describe your early relationship with your baby? - How, if at all, do you think this impacted on your eating disorder? | - Can you think of any specific moments to help describe this? - How did this feel at the time? |
| **Physical health**  *(Difficulties, pregnancy ailments, hormones, weight changes)* | - How would you describe your physical health during the postpartum period? - How, if at all, do you think this impacted on your eating disorder? | - How do you feel this impacted on your thoughts, feelings and behaviour? |
| **Mental health**  *(Anxiety, low mood, post-natal depression)* | - How would you describe your mental health in general during the postpartum period? - How, if at all, do you think this impacted on your eating disorder? | - This sounds really tricky, why do you think you felt this way? - How do you think things might have been different if you didn’t feel this way? |
| **Critical periods**  *(E.g., weaning, returning to work, co-parent returning to work)* | - Were they any parts of the postpartum period that were particularly difficult? - Were they any parts of the postpartum period that felt easier to cope with? | - Why do you feel this was so difficult? - Why do you feel this was helpful? |
| **Other**  *(Other pertinent factors not discussed)* | - Is there anything else, which we have not yet discussed, that you feel was important to your experience of the postpartum period? - Are there any other factors you feel impacted on your eating disorder during the postpartum period? | - In what way do you feel this was important? - How might things have been different if this did not happen? - In what way was this unhelpful? - In what way was this helpful? |
